# Diagnostic value of D-dimer for lower extremity deep venous thrombosis caused by rib fracture: a retrospective study

**DOI:** 10.1186/s13018-023-03997-x

**Published:** 2023-07-20

**Authors:** Lei Feng, Zexin Xie, Xuetao Zhou, Chunjuan Hou, Zheng Liang, Huiqing Lu, Lili Liu, Dongsheng Zhang

**Affiliations:** 1 Department of Cardiothoracic Surgery, The Third Hospital of Shijiazhuang, Shijiazhuang, 050011 Hebei China; 2 Department of Cardiology, The Third Hospital of Shijiazhuang, Shijiazhuang, China

**Keywords:** D-dimer, Fractured ribs, Lower extremity deep venous thrombosis

## Abstract

**Objective:**

This study aimed to investigate the role of D-dimer in the diagnosis of lower extremity deep venous thrombosis (DVT) in patients with rib fractures.

**Method:**

Retrospective analysis was conducted on the clinical data of 499 patients with rib fractures who were admitted to the Third Hospital of Shijiazhuang between October 2020 and September 2021. These patients were divided into the DVT and the non-DVT groups. D-dimer levels were compared between the two groups at 24, 48, and 72 h after the injury. Receiver operating characteristic curves were utilized to evaluate the diagnostic efficacy of dynamically monitoring changes in D-dimer for DVT.

**Results:**

The D-dimer levels in the DVT group were significantly higher than those in the non-DVT group at 24, 48, and 72 h after the injury. The area under the curve values for predicting DVT based on D-dimer level at 24, 48, and 72 h after injury in patients with rib fractures were 0.788, 0.605, and 0.568, respectively.

**Conclusion:**

Detecting D-dimer levels 24 h after the injury can enhance diagnostic efficacy and sensitivity for DVT, thereby reducing the rate of missed diagnoses, which is of great clinical value.

## Introduction

Lower extremity deep vein thrombosis (DVT) is a prevalent complication following traumatic injuries, and its progression can result in life-threatening conditions such as pulmonary embolism (PE) and lower limb dysfunction [1, 2]. Chest trauma ranks as the second leading cause of mortality after head injuries, accounting for approximately 20% of all trauma-related deaths [3]. The incidence and mortality rates associated with chest trauma are primarily determined by the severity of concomitant injuries. Notably, 55%–85% of patients with chest trauma exhibit rib fractures [4].

Patients with rib fractures often experience limited mobility in the immediate aftermath of the injury, leading to alterations in their coagulation function, which can predispose them to DVT development [5]. In the early stages, most individuals with DVT display non-specific symptoms, and the clinical manifestations and signs may not be readily apparent, making timely detection challenging [6]. As the condition progresses, patients may present with symptoms such as lower limb pain and swelling, further complicating treatment options and posing risks [7]. While venography is presently considered the gold standard for diagnosing DVT, it is an invasive procedure that carries the potential risk of inducing DVT itself [8]. Consequently, there is a need for a convenient, safe, rapid, specific, and accurate diagnostic approach in clinical practice. Therefore, the selection of appropriate and effective laboratory indicators to aid in early DVT diagnosis is of utmost importance. Early identification and treatment of DVT are critical for preventing severe complications like PE.

D-dimer, a specific degradation product of fibrinogen, reflects the equilibrium between intravascular coagulation and fibrinolysis [9]. Evaluated D-dimer levels occur when there are abnormalities in coagulation or fibrinolysis within the body [10]. Thus, D-dimer can serve as a sensitive yet non-specific indicator for screening and monitoring DVT occurrence. However, the diagnostic value of D-dimer in patients with rib fractures remains unclear. Consequently, the aim of this retrospective study was to investigate the diagnostic value of D-dimer in identifying lower extremity DVT in patients with rib fractures. This was achieved by comparing D-dimer levels between patients with and without DVT, as well as evaluating the sensitivity and specificity of D-dimer as a diagnostic tool. The findings of this study could provide valuable insights into the development of appropriate thromboprophylaxis strategies for patients with rib fractures, thereby enhancing early diagnosis and prevention of DVT associated with rib fractures, and reducing the rate of missed diagnoses.

## Methods

### Study design and patient recruitment

A retrospective study was conducted to select patients with rib fractures who were admitted to the Department of Cardiothoracic Surgery at Shijiazhuang Third Hospital between October 2020 and September 2021. Patients diagnosed with lower extremity DVT were assigned to the DVT group (n = 90), while those without DVT were assigned to the non-DVT group (n = 409). All included patients underwent routine lower limb venous color Doppler ultrasound on the day of admission. Follow-up examinations or venous angiography were only performed if significant symptoms or special requirements arose. DVT was diagnosed based on color Doppler ultrasound or angiography.

The inclusion criteria were as follows: (1) patients with rib fractures or multiple fractures resulting from external violence; (2) rib fracture diagnosed by a clinician; (3) lower extremity DVT diagnosed by ultrasound or venography after admission; (4) no history of DVT; and (5) patients with normal cognitive and communication skills, capable of completing relevant questionnaires and surveys. The exclusion criteria were as follows: (1) recent treatment with anticoagulant drugs; (2) abnormal coagulation function; (3) history of PE or DVT; (4) history of heart disease, diabetes mellitus, malignancy, vascular disease, or limb paralysis; and (5) patients with psychiatric disorders unable to cooperate with treatment or investigation. All methods were conducted in accordance with relevant guidelines and regulations, adhering to the Declaration of Helsinki. This study received approval from the Ethics Committee of Shijiazhuang Third Hospital (2021–047). As this study employed a retrospective design, written consent was not required.

Sociodemographic information, including sex, age, and body mass index (BMI), was obtained through face-to-face interviews. The type of fracture was obtained from electronic or paper medical records at our center. D-dimer levels were measured using the coagulation analysis system (CS-5100; Sysmex, Kobe, Japan), with the normal reference range for D-dimer being 0–0.55 mg/L. The correlation between D-dimer levels at 24, 48, and 72 h after the injury and disease severity (presence or absence of DVT) was analyzed. Due to variations in the timing of injuries among individuals and the fact that blood tests are typically conducted at a fixed time of 6:00 a.m., it was not feasible to obtain blood samples exactly 24-h post-injury from all enrolled patients. Considering this limitation, the 24-h period encompasses testing within 24 h following the injury.

### Statistical analysis

The data were analyzed using the statistical analysis software SPSS 25.0. Quantitative variables were described using medians (first quartile and third quartile), and comparisons between groups were performed using either the two independent samples t-test or the rank-sum test. Qualitative variables were described using numbers (percentages), and the χ^2^ test was employed for between-group comparisons. Receiver operating characteristic (ROC) curve analysis was conducted to evaluate the predictive ability of D-dimer levels at 24, 48, and 72 h in detecting DVT. *P* < 0.05 was considered statistically significant.

## Results

### Patient characteristics

There were no significant differences in terms of sex, age, body mass index (BMI), and type of fracture between the DVT and non-DVT groups (*P* > 0.05, Table 1).

**Table 1:** Patient baseline demographics and clinical characteristics

Variables	DVT group (n = 90)	Non-DVT group (n = 409)	χ^2^/t	*P*
*Sex*			0.441	0.506
Male	62 (68.9%)	296 (72.4%)		
Female	28 (31.1%)	113 (27.6%)		
*Age (years)*			0.967	0.325
< 60	52 (57.8%)	259 (63.3%)		
≥ 60	38 (42.2%)	150 (36.7%)		
*BMI (kg/m*^*2*^*)*			0.184	0.668
< 28	79 (87.8%)	352 (86.1%)		
≥ 28	11 (12.2%)	57 (13.9%)		
*Type of fracture*			0.038	0.910
Simple rib fracture	32 (35.5%)	143 (35.0%)		
Rib fracture combined with upper limb fracture	18 (20.0%)	81 (19.8%)		
Rib fracture combined with lower extremity fracture	10 (11.1%)	47 (11.5%)		
Rib fracture combined with pelvic fracture	1 (1.1%)	4 (1.0%)		
Rib fracture combined with cone fracture	6 (6.7%)	28 (6.8%)		
Multiple fractures	23 (25.5%)	106 (25.9%)		

*DVT*, deep venous thrombosis and *BMI*, body mass index

### D-dimer levels in patients with rib fractures

D-dimer levels at 24-, 48-, and 72-h post-injury were significantly higher in the DVT group compared to the non-DVT group (*P* < 0.05, Table 2).

**Table 2:** D-dimer levels at 24-, 48-, and 72-h post-injury in both groups (mg/L)

	DVT group	Non-DVT group	*z*	*P*
24 h	7.53 (2.92, 21.98)	1.17 (0.40, 4.35)	− 8.586	< 0.001
48 h	0.15 (0.00, 2.48)	0.03 (0.00, 0.35)	− 4.570	< 0.001
72 h	0.07 (0.00, 1.94)	0.00 (0.00, 0.00)	− 2.911	0.004

*DVT*, deep venous thrombosis

### The diagnostic value of D-dimer in rib fracture-related DVT

**Fig. 1:**
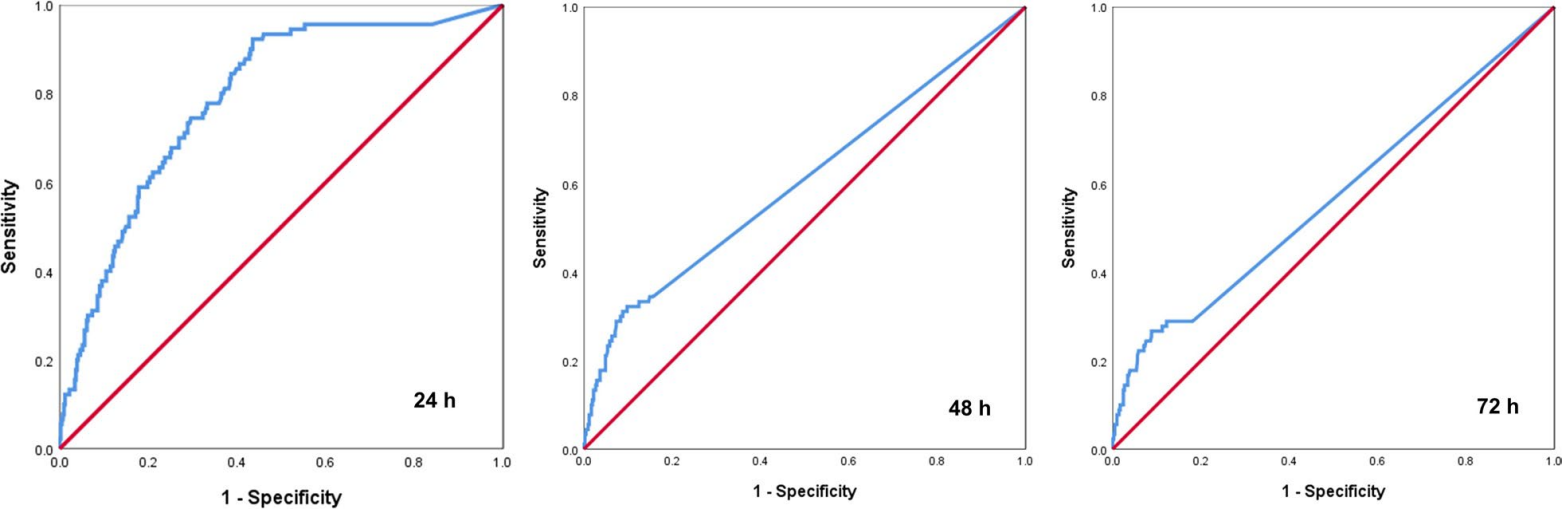
ROC curves of 24-, 48-, and 72-h D-dimer in DVT diagnosis. ROC, the receiver operating characteristic and DVT, deep venous thrombosis

The diagnostic value of D-dimer at 24-, 48-, and 72-h post-injury in identifying DVT in patients with rib fractures was assessed using ROC curves. The area under the curve (AUC) for D-dimer in diagnosing DVT at 24, 48, and 72 h was 0.788, 0.605, and 0.568, respectively (Fig. 1, Table 3).

**Table 3:** ROC parameters

Items	AUC	*P*	95% confidence interval	Sensitivity	Specificity	Cutoff value
24-h DVT	0.788	0.036	0.739–0.838	0.956	0.447	0.935
48-h DVT	0.605	0.036	0.535–0.676	0.344	0.851	1.895
72-h DVT	0.568	0.036	0.498–0.639	0.289	0.844	1.445

*AUC*, the area under the curve and *DVT*, deep venous thrombosis

The positive predictive value of D-dimer at 24 h for VTE in patients with rib fractures was 27.56%, while the negative predictive value was 97.86%. D-dimer levels at 24-h post-injury exhibited high diagnostic efficacy for DVT. To reduce the false-negative rate and achieve high sensitivity while maintaining a reasonable level of specificity, in diagnosing thrombosis in all patients with rib fractures, a diagnostic cutoff value of 0.935 was selected. Values higher than this threshold indicate a higher likelihood of developing lower limb venous thrombosis as a complication of rib fractures. D-dimer levels at 48- and 72-h post-injury had a general diagnostic efficiency for DVT and were not of high diagnostic value.

## Discussion

DVT is a serious complication associated with rib fractures, leading to increased recurrence, disability, and mortality rates [11, 12]. Clinical symptoms of DVT in rib fracture patients, such as lower limb pain and swelling, are often late or subtle, making early detection challenging and resulting in missed diagnoses and delayed treatment. Currently, there is limited research and literature available on accurate predictors of DVT occurrence in rib fracture patients. Therefore, selecting an effective assessment method is of vital clinical significance for the further diagnosis and treatment of secondary DVT in these patients.

Venography, ultrasonography, and Wells scores are commonly used diagnostic methods for DVT [13, 14]. However, venography carries risks of adverse reactions and is costly. Lower limb vascular ultrasonography is a non-invasive method that provides information on thrombus location, the intensity of thrombus echo, and blood flow imaging, but it may have limitations in detecting small or deep lesions, leading to a certain rate of missed diagnosis and misdiagnosis [15]. The specificity of lower limb vascular ultrasonography in diagnosing proximal DVT is reported to be above 98%, with a sensitivity of up to 95% [16]. However, routine ultrasound has poor image quality and low diagnostic efficiency for early DVT, relying on the subjective experience of the physician [17]. The Wells score is commonly used as a reference indicator for clinical diagnosis but requires complementary evaluations with other examinations.

D-dimer is primarily a specific degradation product generated through fibrinolytic enzyme hydrolysis of fibrin monomers that are cross-linked by activated factors. It reflects the coagulation and concurrent fibrinolysis state in the body and is frequently used in the diagnosis and evaluation of venous thromboembolism. Quantitative detection of D-dimer is useful for identifying patients at risk of DVT development [18, 19]. In this study, we investigated the dynamic monitoring of D-dimer in rib fracture patients with and without DVT. Among the 90 patients who developed DVT after rib fractures, D-dimer levels at 24-, 48-, and 72-h post-injury were significantly higher compared to those who did not develop DVT, demonstrating statistical significance (*P* < 0.05).

To further clarify the diagnostic value of D-dimer in predicting DVT in patients with rib fractures, we constructed ROC curves for diagnosing DVT based on the D-dimer levels at 24-, 48-, and 72-h post-injury, respectively. The results revealed that D-dimer levels at 24-h post-injury had the highest diagnostic efficiency for DVT, with an AUC value of 0.788 (*P* = 0.036, 95% CI 0.739–0.838). This indicates that D-dimer at 24-h post-injury can effectively discriminate between patients with and without DVT. The sensitivity of the D-dimer at 24 h was 0.956, while the specificity was 0.447. The positive predictive value was 27.56%, and the negative predictive value was 97.86%. In contrast, D-dimer levels at 48- and 72-h post-injury showed a moderate diagnostic efficiency for DVT. The AUC values of D-dimer levels at 48 and 72 h were 0.605 (*P* = 0.036, 95% CI 0.535–0.676) and 0.568 (*P* = 0.036, 95% CI 0.498–0.639).

Based on the analysis, we determined an optimal cutoff value for D-dimer of 0.935. Values above this cutoff suggest a higher likelihood of DVT occurrence. Therefore, when the D-dimer value exceeds 0.935, even if a lower limb venous color ultrasound does not indicate thrombosis formation, initiation of regular anticoagulation treatment is recommended. However, for patients with D-dimer values below this cutoff, routine prophylactic anticoagulation treatment may not be necessary. Instead, encouraging early mobilization and lower limb exercise can be sufficient. D-dimer levels serve as an effective tool for diagnosing DVT because they reflect abnormal coagulation function in the body. In the hypercoagulable state or when secondary fibrinolysis is overactivated, D-dimer levels specifically increase. In cases of chest trauma or complex injuries, the body undergoes varying degrees of bleeding, leading to a hypercoagulable state and the formation of thrombosis [20, 21]. Elevated D-dimer levels may stimulate the inflammatory process and the release of pro-inflammatory cytokines, contributing to the development of DVT [22, 23]. Therefore, early monitoring of D-dimer can provide predictive information on DVT occurrence and guide appropriate preventive measures [24, 25].

Thromboelastography (TEG) has been shown to be useful in diagnosing traumatic hypercoagulable disorders, but its application is limited due to the high cost and limited availability of equipment [26]. D-dimer, on the other hand, offers the advantages of simplicity and cost-effectiveness and can serve as a complementary diagnostic tool. Therefore, measuring D-dimer levels within 24 h after the injury is clinically significant. However, it is important to note that elevated D-dimer levels can also be observed in various other conditions, such as pregnancy, trauma, postoperative states, inflammatory states, renal diseases, stroke, myocardial infarction, disseminated intravascular coagulation, and tumors [27]. When using D-dimer as a diagnostic marker for DVT, it is crucial to develop detailed exclusion criteria and consider combining it with other effective plasma or serum markers.

In summary, patients with rib fractures who are at risk of developing DVT may exhibit abnormal D-dimer levels. The research indicated that D-dimer levels at 24-h post-injury have diagnostic value for the occurrence of thrombosis. In clinical practice, it is recommended to promptly test D-dimer levels in patients with rib fractures and retest within 24 h. Targeted prevention and treatment should be implemented for patients with significantly abnormal D-dimer values. Emphasizing monitoring indicators, standardizing rescue processes, and initiating early prophylactic anticoagulation therapy can improve treatment success rates [28–30]. However, due to the small sample size and the complexity and criticality of rib fracture patients caused by chest trauma, it is important to further investigate the evaluation system for DVT complications in rib fracture patients. Large-sample retrospective and cohort studies are needed to explore the relationship between patient prognosis and various abnormal indicators. This research has significant implications for clinicians in the early evaluation and treatment of DVT in rib fracture patients. It should be noted that although D-dimer levels can be elevated in cases of pulmonary embolism (PE), rib fracture-related PE events do not appear to be completely linked to the occurrence of DVT [31]. Local inflammation, hidden vascular damage, low-flow state, protein C consumption, hypercoagulable state, and adrenal responses after trauma can contribute to vascular endothelial inflammation, the production of circulating adhesion molecules, local thrombosis, and rapid occlusion, potentially explaining the “in situ” formation of PE [32].

## Data Availability

The datasets used and/or analyzed during the current study are available from the corresponding author on reasonable request.

## Abbreviations

DVT: Deep venous thrombosis
ROC: Receiver operating characteristic
PE: Pulmonary embolism
BMI: Body mass index
AUC: Area under the curve
TEG: Thromboelastography
